# A robust linkage map of the porcine autosomes based on gene-associated SNPs

**DOI:** 10.1186/1471-2164-10-134

**Published:** 2009-03-27

**Authors:** Rikke KK Vingborg, Vivi R Gregersen, Bujie Zhan, Frank Panitz, Anette Høj, Kirsten K Sørensen, Lone B Madsen, Knud Larsen, Henrik Hornshøj, Xuefei Wang, Christian Bendixen

**Affiliations:** 1 Department of Genetics and Biotechnology, Faculty of Agricultural Sciences, Aarhus University, PO Box 50, 8830 Tjele, Denmark

## Abstract

**Background:**

Genetic linkage maps are necessary for mapping of mendelian traits and quantitative trait loci (QTLs). To identify the actual genes, which control these traits, a map based on gene-associated single nucleotide polymorphism (SNP) markers is highly valuable. In this study, the SNPs were genotyped in a large family material comprising more than 5,000 piglets derived from 12 Duroc boars crossed with 236 Danish Landrace/Danish Large White sows. The SNPs were identified in sequence alignments of 4,600 different amplicons obtained from the 12 boars and containing coding regions of genes derived from expressed sequence tags (ESTs) and genomic shotgun sequences.

**Results:**

Linkage maps of all 18 porcine autosomes were constructed based on 456 gene-associated and six porcine EST-based SNPs. The total length of the averaged-sex whole porcine autosome was estimated to 1,711.8 cM resulting in an average SNP spacing of 3.94 cM. The female and male maps were estimated to 2,336.1 and 1,441.5 cM, respectively. The gene order was validated through comparisons to the cytogenetic and/or physical location of 203 genes, linkage to evenly spaced microsatellite markers as well as previously reported conserved synteny. A total of 330 previously unmapped genes and ESTs were mapped to the porcine autosome while ten genes were mapped to unexpected locations.

**Conclusion:**

The linkage map presented here shows high accuracy in gene order. The pedigree family network as well as the large amount of meiotic events provide good reliability and make this map suitable for QTL and association studies. In addition, the linkage to the RH-map of microsatellites makes it suitable for comparison to other QTL studies.

## Background

Genetic linkage maps are essential tools for locating genes and quantitative trait loci (QTLs) that control important traits. The first linkage map covering all 18 autosomes of the pig was published in 1995 [1], followed by a large map containing approximately 1,200 markers [2]. These maps were primarily constructed on the basis of anonymous microsatellites and restriction fragment length polymorphism (RFLP) markers [1-3]. Other marker types including amplified fragment length polymorphisms (AFLP) and single nucleotide polymorphisms (SNPs) have been added to online versions of the maps [4].

SNP-based genetic variation is found with high density throughout the genome. Efficient technologies have been developed, which allow for highly parallel and cost efficient genotyping, SNPs have therefore become the markers of choice for genetic mapping. This makes SNP maps highly suitable for association studies, fine mapping of QTLs as well as haplotype determination. Moreover, to identify the genes underlying monogenic and quantitative traits, it is an advantage if the maps are based on gene-associated markers, such as genic SNPs. Genic SNPs, whether they are located in coding or in 5' and 3' untranslated regions are more likely to cause a functional change than those that occur outside genes [5]. Due to linkage disequilibrium intergenic SNPs closely linked to causative mutations in genes of interest can be of value [6]. The use of gene-associated SNPs implies an increased knowledge of the genomic region of interest and facilitates the possibility of identifying candidate genes and the actual genes that underlie the trait. At present, more than one million porcine expressed sequence tags (ESTs) are available [7], and tools to evaluate and select candidate SNPs in coding regions for application as genetic markers have been developed [8].

The pig genome has more conserved synteny with human than with mouse [9] and many of the porcine ESTs are orthologs of parts of human genes. Human-pig comparative maps based on ESTs exclusively [10] as well as ESTs in combination with bacterial artificial chromosome (BAC) end sequences [11] have been constructed using the INRA-Minnesota porcine Radiation Hybrid panel, IMpRH_7000 _[12]. This panel is a valuable tool for map refinement as it allows good precision in mapping. Moreover, a highly continuous BAC map of the pig genome has been developed [13]. Gene annotation, map positions and order of SNP markers developed from ESTs can be verified using the information derived from the BAC maps or from genes and sequences mapped in humans. In addition, comparison to human genes is a tool to link characteristics to causative genes in future QTL studies. Especially for insufficiently described regions, the comparative mapping provides the possibility of identifying central genes through programmes like GeneDistiller [14].

Previously, SNPs have been used for mapping only selected parts of the pig genome [15-17]. Here we present a linkage map covering all 18 porcine autosomes. The map is based on a large number of offspring and a high number of meioses that is suitable to establish gene order and genetic distances. In addition to application in gene-based genome-wide QTL and association studies, a map based on SNPs developed from EST sequence data is of value for the porcine genome project, providing information for validation of assembly and ordering of sequenced regions. Furthermore, this SNP map is useful as an anchoring map for future dense maps based on data from the PorcineSNP60 Genotyping BeadChip [18] (WG-410-1001-PRE, Illumina) due to marker overlap.

## Results

### SNP selection and genetic map

The 4,600 exons, from which the SNPs were identified, were distributed on all 22 human chromosomes. A total of 709 SNPs were initially detected in sequence alignments of the gene associated amplicons derived from EST and shotgun sequences. If a SNP segregated in at least one of the 12 Duroc boar families it was selected for further analysis. Of these, 506 SNPs were annotated prior to the mapping process. The remaining 203 SNPs were rejected either because of failure in assay design or low call rate in the genotyping assays. During the annotation six SNPs were discarded due to similarity and artefact problems. Six SNPs (Additional file 1) were not similar to any known gene or EST in the human genome and were mapped as porcine EST-based SNPs (designated as P followed by four digits).

After annotation of the SNP surrounding sequence the allelic structure of the SNPs were analysed. A between-family analysis showed differences that could only be accounted for by genotyping mistakes. Differences in how the resulting clusters were interpreted emerged because it was not always clear which cluster was heterozygote and which was homozygote if only two clusters were present. The data set was therefore reduced to 481 SNPs.

The two-point analysis resulted in 18 large linkage groups (LOD > 75) each assigned to one of the porcine chromosomes using the comparative map from INRA [19]. In addition, seven small linkage groups with an average of three SNPs in each were produced. Comparative mapping indicated that the largest of these, which comprised five SNPs located in the genes *USP24*, *EIF2B3*, *OMA1*, *CPT2*, and *GPX7*, was associated with the distal part of porcine chromosome 6 (*Sus scrofa *chromosomes, SSC 6). A few individual SNPs were assigned to linkage groups based on slightly lower LOD scores (LOD > 55) together with comparative association, whereas the remaining small linkage groups and singletons were rejected as a consequence of low linkage ability. Finally, a total of 462 SNPs, distributed on 440 different genes, and six EST sequences were mapped to the 18 porcine autosomes (SSC 1 to 18).

**Figure 1:**
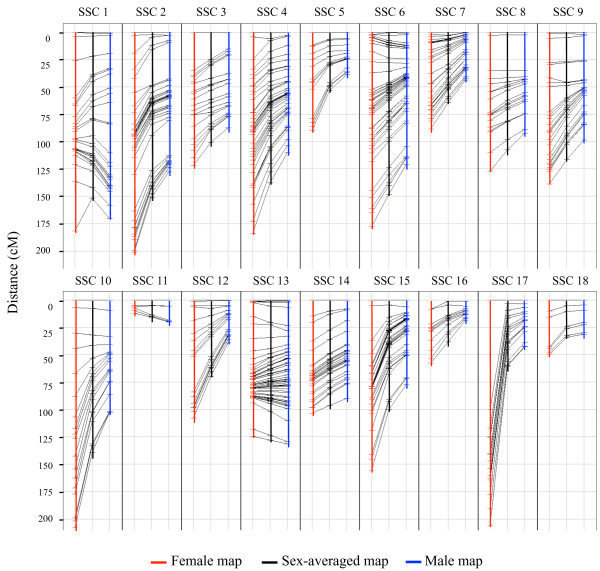
**Female, sex-averaged and male linkage maps of the 18 porcine autosomes**. Each chromosome is presented showing the relation in distance between the markers on the female, sex-averaged and male linkage maps.

The sex-averaged map covered 1,711.8 cM with an average SNP distance of 3.94 cM, whereas the female map covered 2,336.1 cM and the male map 1,441.5 cM (Table 1). The chromosome length of the sex-averaged maps ranged from 15.7 cM for SSC 11 and 151.4 cM for SSC 1, and the number of SNPs on each chromosome map varied between six and 57 on SSC 11 and SSC 13, respectively (Table 1). A comparison of the sex-averaged, female and male maps is illustrated in Figure 1. The exact SNP position on these maps as well as information regarding MAF in the sows, number of meioses and heterozygous sires are indicated in Additional file 2.

**Table 1:** Length of the sex-averaged, female and male linkage maps.

**SSC^1^**	**Sex-averaged** **length (cM)**	**Female** **length (cM)**	**Male** **length (cM)**	**SNP^2 ^count**	**Average distance (cM)**
1	151.4	181.7	170.2	26	5.82
2	150.7	201.2	127.7	41	3.68
3	100.5	121.9	87.3	20	5.03
4	137.6	183.8	109.9	40	3.44
5	53.4	89.7	38.4	14	3.81
6	148	177.9	121	50	2.96
7	64.2	88.5	43.7	29	2.21
8	107.4	126.9	92.4	19	5.65
9	116.6	138.9	98.4	27	4.32
10	139.5	208.1	102.5	20	6.98
11	15.7	12.1	20	6	2.62
12	69.8	107.9	39.1	17	4.11
13	128	124.5	131.3	57	2.25
14	96.3	103.3	89.9	28	3.44
15	100.3	156.2	77.4	31	3.24
16	38.1	57.6	18.5	13	2.93
17	60.2	205.8	42.6	17	3.54
18	34.1	50.1	31.2	7	4.87

**Total**	**1711.8**	**2336.1**	**1441.5**	**462**	**3.94**

^1 ^*Sus scrofa *chromosome ^2 ^Single nucleotide polymorphisms

**Figure 2:**
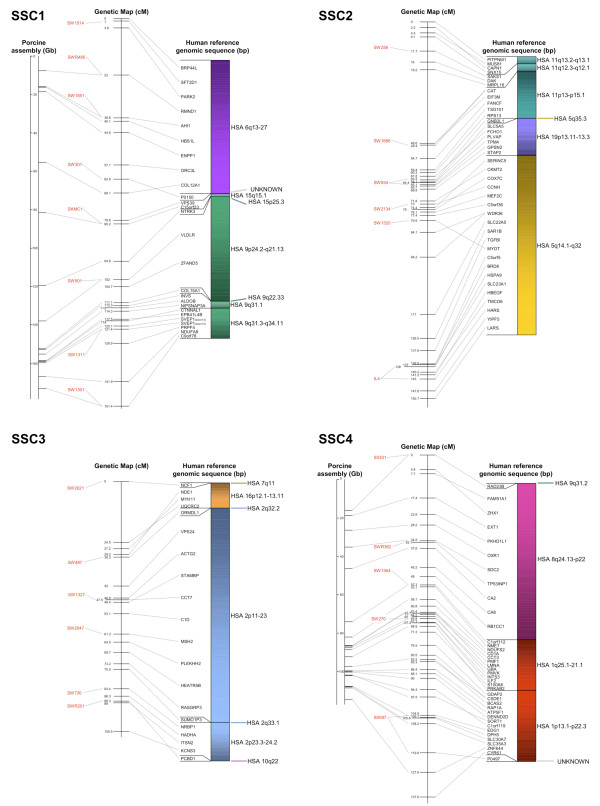
**Averaged-sex linkage maps of porcine chromosome 1 to 4**. Each linkage map (centre) is presented with linked microsatellite markers and if known the BAC assembly map positions (left), and the human reference sequence (right). The genes are presented in the order of the human genome and a link between position on the porcine genetic map and human reference and BAC assembly sequence is indicated by a grey line. The colour scheme for the human chromosomes is found on Figure 6.

**Figure 3:**
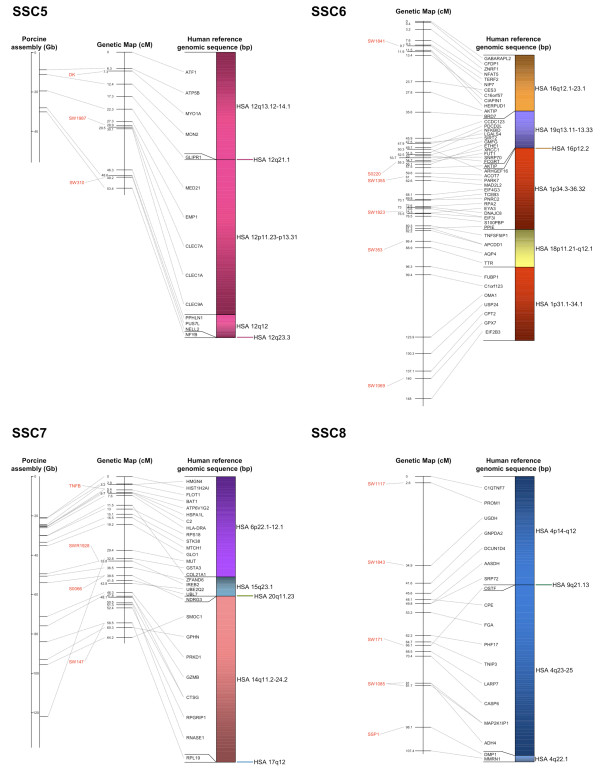
**Averaged-sex linkage maps of porcine chromosome 5 to 8**. Each linkage map (centre) is presented with linked microsatellite markers and if known the BAC assembly map positions (left), and the human reference sequence (right). The genes are presented in the order of the human genome and a link between position on the porcine genetic map and human reference and BAC assembly sequence is indicated by a grey line. The colour scheme for the human chromosomes is found on Figure 6.

**Figure 4:**
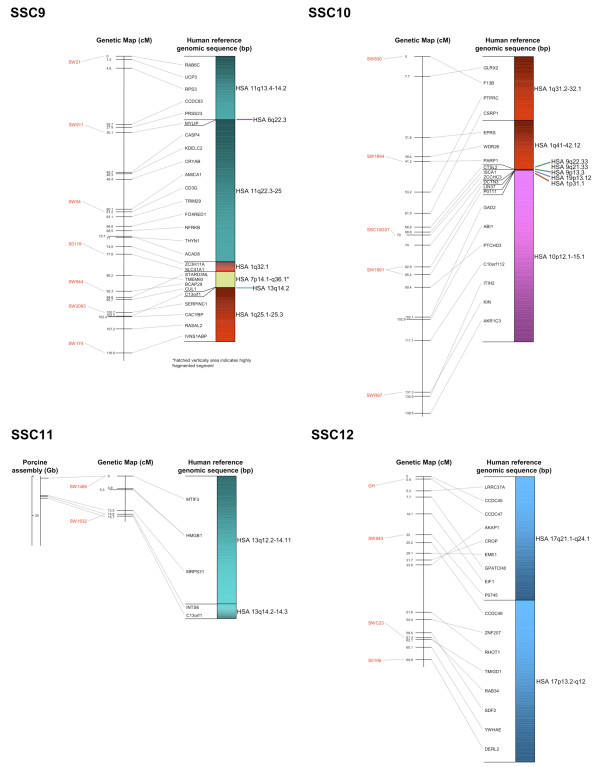
**Averaged-sex linkage maps of porcine chromosome 9 to 12**. Each linkage map (centre) is presented with linked microsatellite markers and if known the BAC assembly map positions (left), and the human reference sequence (right). The genes are presented in the order of the human genome and a link between position on the porcine genetic map and human reference and BAC assembly sequence is indicated by a grey line. The colour scheme for the human chromosomes is found on Figure 6.

**Figure 5:**
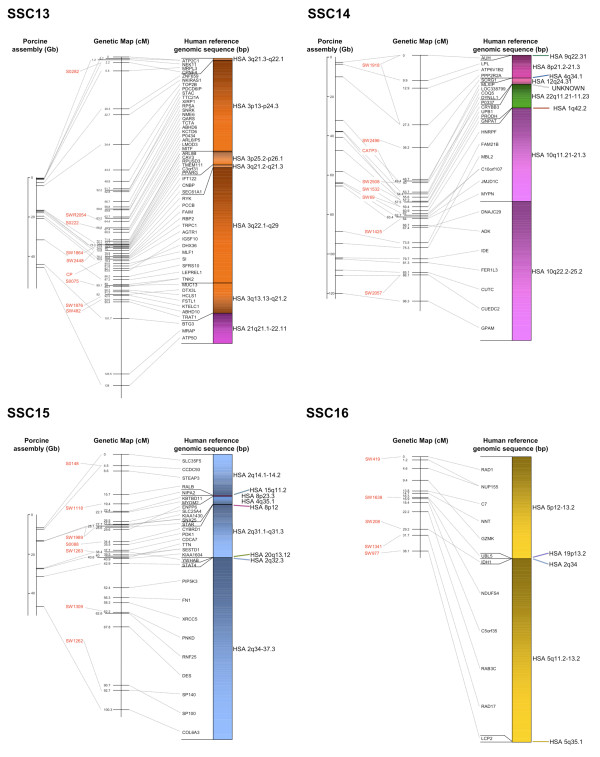
**Averaged-sex linkage maps of porcine chromosome 13 to 16**. Each linkage map (centre) is presented with linked microsatellite markers and if known the BAC assembly map positions (left), and the human reference sequence (right). The genes are presented in the order of the human genome and a link between position on the porcine genetic map and human reference and BAC assembly sequence is indicated by a grey line. The colour scheme for the human chromosomes is found on Figure 6.

**Figure 6:**
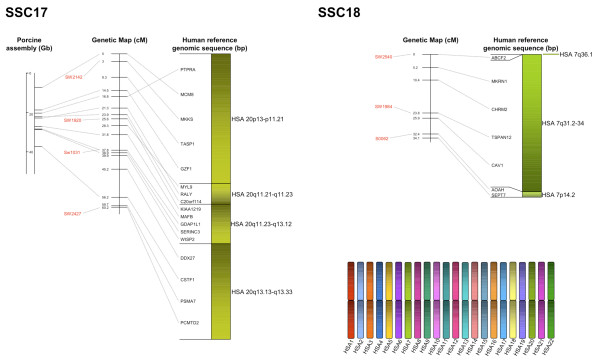
**Averaged-sex linkage maps of porcine chromosome 17 and 18**. Each linkage map (centre) is presented with linked microsatellite markers and if known the BAC assembly map positions (left), and the human reference sequence (right). The genes are presented in the order of the human genome and a link between position on the porcine genetic map and human reference and BAC assembly sequence is indicated by a grey line. The colour scheme for the human chromosomes is found on Figure 6.

The BAC assembly sequence of each chromosome is shown in relation to the genetic linkage maps as well as human reference sequences (Figures 2, 3, 4, 5 and 6). Microsatellites linked to evenly spaced markers using the IMpRH_7000 _on each SSC are also presented in the figures.

### SNP localisation

A total of 97.8% of the sequences was matched to genes by Blast analysis against the refGene database. The remaining sequences were not matched to the available human exon information and as a consequence the genic status of these were classified as unknown (Table 2).

**Table 2:** Single nucleotide polymorphism (SNP) status.

**SNP information**	**# SNP**
3'-UTR	52
5'-UTR	3
Synonymous	121
Non-synonymous	53
Intragenic	223
Unknown	10

**Total**	**462**

### Validation through comparative mapping

Comparison of our linkage map and the RH-based comparative map from INRA indicated a high correlation with the expected pattern of gene localisation. The marker gene order of the genetic map was compared to the order of previously physically and genetically mapped porcine genes. Only one of the genetically mapped genes *F13B *differed from previous results by being mapped to chromosome SSC 10 instead of SSC 4 (Additional file 1). Some genes or regions showed rearrangements on the porcine map compared to the expected order. To verify the linkage mapping the SNP-containing sequences in question were mapped onto the IMpRH_7000 _panel (Additional file 1).

For a better comparison to the microsatellite map evenly spaced SNP-containing sequences were mapped onto the IMpRH_7000 _panel (Additional file 1). A few rearrangements in the microsatellite order in relation to our SNP map were found.

Comparisons to the available chromosomes of the BAC assembly indicated a high similarity with the marker order. A total of 119 SNP sequences were found to match the chromosomes SSC 1, 4, 5, 7, 11, 13, 14, 15 and 17 by Blast analysis against the BAC assembly (Additional file 1). The relation between distance of markers on the assembly map and the genetic map was about 1:1 within each chromosome (Figures 2, 3, 4, 5 and 6). However, there was no obvious relation between the lengths of the BAC assembly and the genetic map between chromosomes indicating a difference in recombination rate between the individual chromosomes.

Markers were mapped to previously reported syntenic regions [19] except for three large segmental regions that was not identified in our study, two at SSC 1 from human chromosomes (*Homo sapiens*, HSA) 18 and 14, and one at SSC 5 equal to HSA 22q12-qter. None of the genes that were located in the linkage group matching SSC 12 have previously been mapped in pig and hence the verification and orientation of this chromosome relies on the comparative map and linkage to microsatellites located on SSC 12. All the genes showed homology to HSA 17p13.3-q24 indicating that these genes most likely should be located on SSC 12. This was further indicated by IMpRH_7000 _mapping of five SNP-containing sequences belonging to the SSC 12 linkage group (Additional file 1 and Figure 4).

Through linkage to the RH-mapped microsatellites [20] it was confirmed that the SNPs covered a large part of the autosome. However, by comparison to the linkage map [2] that relates to the RH-map, about 20% seems to be missing. The map presented here is shorter due to missing markers on parts of SSC5, SSC7, SSC11, SSC12, SSC16 and SSC18.

## Discussion

The map presented here is the first map of all 18 porcine autosomes based on gene-associated SNPs and it contains 330 not previously located genes. The positions of 81 SNP-containing sequences representing these genes were confirmed by Blast analysis against the newly assembled BAC porcine sequence (Additional file 1).

A total of 456 gene-associated and six porcine EST-based SNPs identified from re-sequenced exons were mapped to the 18 porcine autosomes. The main selection of the SNPs was unbiased as they were found randomly in the amplicons containing the EST sequences, which were distributed across the 22 human autosomes. Almost all SNPs showed heterozygosity in the sires except 14 SNPs that were included because of interest to other projects. As the SNPs were selected on linkage ability with a very high two-point LOD score (>55) the number of informative meioses is expected to be high. In the present study it ranges from 401 to 6,898 allowing us to calculate a very robust map. The family comprised Duroc sires crossed with Danish Landrace/Danish Large White sows and as the SNPs were selected from alignments of re-sequenced exons in the 12 Duroc sires this implies that most of the informative meioses arose from this population. For the sow population we considered instead the minor allele frequency (MAF) of each SNP which gave us an idea of the distribution between male and female meiosis. The analysis did not indicate that the difference in meiosis numbers affected the results as the number generally was very high (Additional file 2).

The length of the averaged-sex linkage map was calculated to be 1,711.8 cM, the female map was 2,336.1 cM and the male map was 1,441.5 cM. As described in the result section when comparing to the map of Rohrer and colleagues [2] our map is about 20% shorter. The animal material they used to create their linkage map is much smaller than the material used here, which might influence the recombination rates at the ends of the chromosomes and thereby overestimate the length. We see this phenomenon on the female map when the number of meioses is low like on SSC 17p where the MCM8 marker is positioned 100 cM from MKKS (Figure 1). The first whole-genome map estimated the female map to be about 21 Morgan (M) and the male map around 16.5 M [1]. When these female and male maps are compared a recombination ratio of 1.3:1 is found. Another estimation of the ratio suggests the recombination ratio to be 1.55:1 [3]. In our case the ratio was even higher, approximately 1.65:1, resembling the human recombination ratio of 1.7:1 [21].

When considering each chromosome map of the female and male only three chromosomes separate from the rest (Figure 1). The SSC 11 and the SSC 13 differ from the rest by having a longer male map. Regarding SSC 11, this is in accordance with previous work where a longer p-arm of the SSC 11 male map was found [22]. However, these authors also reported that the complete male map of SSC 11 was shorter than the female map, which could be the case in the present study too if the entire map of SSC 11 was available. On SSC 1 there is a clear difference in how the distance of the markers on the female and male maps varies across the entire chromosome as reported previously [23]. However, the female map presented here is longer than the male map, which is due to a single marker (C9orf78) positioned at the telomeric end of SSC 1q. The MAF found in the sow population of this marker is low leading to few female informative meioses, which can explain the high recombination rate between the markers NDUFA8 and C9orf78 on the female map.

The linkage analysis showed that most synteny groups were present and only few single gene and micro rearrangements were found. Three synteny regions at SSC 1 were not represented on the linkage map, i.e. the HSA 14q21 region and both extremes of HSA 18. The HSA 22q12-qter, which mapped to SSC 5 [19], was also not present on our map. In addition to the absence of these regions, segments from four human chromosomes were missing on SSC 2 (HSA 1), SSC3 (HSA 9) and SSC 17 (HSA 4 and 8) when comparing to the comparative segments identified by Meyers and colleagues [11]. These regions were missing due to the fact that no SNPs were identified in exons representing these genomic areas. However, on SSC 3, 7, 8, 9, 10, 15 and 16 the map presented here contains nine additional regions. In addition, the telomeric ends of SSC 3 and 4 were divided in more segments than the previous comparative map [11], though in general the present map is divided in fewer segments, probably due to fewer markers. Apart from this, the similarity concerning conserved synteny between these two maps is very high.

All single gene rearrangements and SNPs denoting porcine ESTs were analysed to verify the linkage order. A total of ten genes and six ESTs were mapped onto the IMpRH_7000 _panel and in all cases the location was confirmed (Figures 2, 3, 4, 5 and 6). The six ESTs were all mapped to the human genomic sequence. In three cases the location matched the expected synteny group but for P0497, P0150 and P0337 the human homologous region could not be determined.

The nine new regions discussed above were mapped onto the IMpRH_7000 _panel to confirm their locations. These regions refer to the following genes: *PCBD1 *from HSA 10q22.1 on SSC 3; *NDRG3 *and *RPL19 *from HSA 20q11.23 and HSA 17q12, respectively, on SSC 7; *OSTF1 *from HSA 9q21.3 on SSC 8; *MYLIP *from 6p22.3 on SSC 9; *LIN37 *from HSA 19p13.12 on SSC 10; *YWHAB *from HSA 20q13.12 on SSC 15 and finally *IDH1 *and *UBL5 *from HSA 2q34 and HSA 19p13.2, respectively, on SSC 16. None of these genes/human regions have previously been mapped to these porcine chromosomal locations [11], but *NDRG3*, *RPL19 *and *YWHAB *were confirmed by Blast of the SNP-containing sequence against the recently assembled porcine map [13]. Moreover, all gene locations and orders in relation to other markers were confirmed by linkage to the microsatellite RH-map [20]. Most of the single gene rearrangements were located in regions between larger synteny groups, which also support the calculated marker order on this genetic map.

Of the 440 genes and ESTs located on the map, a total of 110 genes have previously been mapped either by linkage or physically and all of these except one were mapped to the expected chromosome. The *F13B *gene located at HSA 1q31.3 deviated as it was formerly mapped by linkage analysis to SSC 4 [24], but as this fragment of the human genome is known to correspond to a region on SSC 10 [10] the difference is highly likely. The linkage result of *F13B *to SSC 10 was confirmed by mapping the gene onto the IMpRH_7000 _panel.

Only two previously physically mapped genes were ordered differently than expected. The first was the *TPM4*, which was mapped to SSC 2q24-q29 [25]. In the present study it was positioned further up on the linkage map than *MEF2C *on SSC 2q21-q22 (Figure 2). On the comparative map of this region, only *TPM4 *from HSA 19 is located among genes from HSA 5 [19] which indicates that our positioning of the *TPM4 *gene among other genes from HSA 19 might be correct. The other gene that was located at a different position than expected was *FUT1 *from SSC 6q11 (Figure 3). This gene was previously cytogenetically mapped closer to the centromeric region than the genes from SSC 6q12 [26]. In our analysis the gene was located more distal, close to the *XRCC1 *gene from the same human chromosomal region on HSA 19q13.31. The *XRCC1 *gene has previously been mapped to SSC 6q12-22 [27] which might be the actual position of the *FUT1 *gene as well.

A total of 81 genes were matched by Blast analysis of SNP-containing sequences against the PreEnsembl porcine BAC sequence. For nine short regions containing one to three markers on the chromosomes, minor rearrangements of the gene order occurred (marked in bold in Additional file 1). Within these regions of minor rearrangements the distance between the markers was small both on the linkage map and on the BAC assembly. The discrepancies could be caused by rearrangements in the BAC assembly of the porcine chromosomes or deviations on the genetic map, due to missing segregations in some boar families despite the SNP network resulting from sows having litters with more than one boar. However, these areas should be subject to further investigation.

This SNP map forms the basis for ongoing studies on QTLs for meat quality, growth, osteochondrosis, lung diseases and other traits. An advantage of the map is the ability to compare to previously reported QTL studies made on microsatellite markers as well as the fact that 138 of the SNPs is represented on the new Illumina PorcineSNP60 Genotyping BeadChip [18]. The actual SNP overlap can be identified through the PorcineSNP60 name (Additional File 2). Therefore, the map can function as an anchoring map for future maps created by use of Bead Chip technology. Furthermore, new SNPs can be added to this version of the map for fine-mapping.

## Conclusion

Of the 462 genetically mapped SNPs, a total of 330 genes were located for the first time on the porcine chromosomes. The linkage map of the porcine genome reported here is based on a large number of meioses providing high accuracy in the relative ordering of genes and in the estimate of genetic distances. Only few discrepancies were observed between the present and previous studies. As this map was calculated on gene-associated SNPs the generated SNP map will be valuable for further QTL and association studies of porcine traits with importance for health and production as well as for verifying the genome assembly. In addition, it will be useful as a framework map.

## Methods

### The family material

A two-generation pedigree was produced by crossing 12 Duroc boars from the Danish breeding program (DanAvl) with 703 Danish Landrace/Danish Large White sows from three different Danish pig production herds. Boars unrelated to at least the great-grandparent level were chosen based on selection index. Some of the sows were related as aunts or sisters. A total of 1,079 litters with an average of ten piglets per litter were produced and offspring from all boars were represented in all three stable environments. A total of 150 disease and phenotypic traits were registered for each pig. The calculations for this genetic map were performed on 236 sow families since these sows had litters with more than one boar. This provided the possibility for generating accurate genetic maps, even though a close network of SNPs segregating in some of the boar families was missing. Blood samples from the sows were collected in the stables. Tissue samples from tenderloin (*psoas major*) were collected from the piglets and boars at processing. Liver samples were collected from piglets that died before slaughtering. Genomic DNA was isolated from all specimens by treatment with proteinase K followed by sodium chloride precipitation [28].

### Exon re-sequencing and primer design

A total of 4,600 candidate exons for SNP detection were selected from alignment of porcine EST sequences from the Sino-Danish Pig Genome Sequencing Project [9]. The human reading frame information was used to capture the exons and the surrounding sequence [29]. PCR primer pairs were designed using Primer3 [30] and purchased from DNA Technology (Aarhus, Denmark). The exons were PCR-amplified in a reaction mixture with a total volume of 10 μl containing 0.25 mM dNTP, 2.5 mM MgCl_2_, 10 × Buffer II Applied Biosystems, 0.5 U AmpliTaq Gold DNA polymerase (PE, Applied Biosystems, Foster City, California), 5 pmol primer and 20–100 ng of genomic DNA. The cycling conditions were: 94°C for 12 minutes; 10 cycles at 94°C for 15 seconds, 65°C for 15 seconds with a touchdown of 0.5°C per cycle, and 72°C for 15 seconds, followed by 30 cycles with an annealing temperature reaching 60°C, and finally an extension step at 72°C for 7 minutes and storage at 4°C.

DNA sequencing of the 12 Duroc boars was performed using BigDye Terminator v.3.1 Cycle Sequencing with AmpliTaq DNA polymerase FS (ABI PRISM™ Genetic Analyzer Model 3730xl, PE, Applied Biosystems). Automated SNP detection was performed using PolyPhred v4.05 [31] and candidate SNPs were selected after visual inspection of the respective chromatograms. For mapped SNPs one sequence for each allele were submitted to GenBank. Information regarding the ID, primers and sequences surrounding the SNP, sequence ID and GenBank Accession numbers are listed in Additional file 2.

### Genotyping

The SNPs were genotyped in the family material either by TaqMan oligo-displacement assay (Assay-by-Design) or SNPlex Genotyping System (PE, Applied Biosystems). Reactions were carried out according to manufacturer's protocols, using dried DNA in optical 384-well plates. The TaqMan assay fluorescent signals were detected on an ABI PRISM SDS 7900 HT Sequence Detection System. Results were analysed in the SDS 2.1 software for allelic discrimination. The SNPlex assay signals were detected on an automated DNA sequencer (ABI PRISM™ Genetic Analyzer Model 3730xl) and the results were analysed using the GeneMapper software v3.7. Most SNPs that were selected for the map were heterozygous in at least one sire. A total of 14 SNPs were only heterozygous in the sow population but included in the analyses because they were expected to hold interesting characteristics in relation to other projects. The number of informative meioses, the MAFs of the SNPs in the sow population and the number of heterozygous sires for each SNP was calculated and for each SNP the applied assay is given (Additional file 2).

### Annotation of the SNP-containing genes

Sequences surrounding the SNPs were subjected to blast analysis on a DeCypher FPGA computer using the accelerated BLASTNH algorithm (Timelogic/Active Motif). To get the human homologous genes the refGene database retrieved from the UCSC Genome Browser database was used [29,32]. The hg18 (NCBI build36.1) exon FASTA sequences were retrieved from the UCSC Table Browser [33,34] and used for SNP annotation. For SNP-containing sequences with no match to any known genes the representative EST sequence was used. For all sequences the cytogenetic location and physical position of the human homologue were obtained from Ensembl [35]. Information on SNP-containing genes is available in Additional file 2.

### Linkage analysis

The averaged-sex, female and male linkage maps were calculated using CRIMAP v. 2.50, which is a revised version of CRIMAP v. 2.4 [36] modified by Jill Maddox and Ian Evans (Jill Maddox, University of Melbourne, pers. comm.). Pair-wise linkage analysis was performed with the TWOPOINT function. The SNPs were initially divided into linkage groups based on the LOD threshold (LOD > 75). However, a few SNPs were added to the linkage groups with a LOD > 55. The BUILD option was used to determine the best order of the SNPs in each linkage group. Subsequently, multipoint linkage analysis was performed to determine the most significant position of the SNPs in each linkage group by sequential insertion of SNPs using the ALL option. The FLIPS option was used to ensure correctness of the order. The order of markers in each linkage group was confirmed using the flips4 option. The comparative map between human and porcine segments from INRA [19] was used as an initial guide to link each linkage group to the 18 porcine autosomes.

### Literature study of gene position

A literature search was conducted to find the positions of the SNP-containing genes previously located on the porcine autosomes. This was done to validate the marker order that was calculated on our map. The previously mapped genes (listed according to the map order) are: *PARK2 *[37], *ENPP1 *[38], *NTRK3 *[39], *VLDLR *[40], *COL15A1 *[41], *ALDOB *[42], *C9orf78 *[43], *CAPN1 *[25], *CAT *[44], *RPS13 *[45], *GNB2L1 *[46], *TPM4 *[25], *CKMT2 *[47], *MEF2C *[48], *SLC22A5 *[16], *SAR1B *[49], *HBEGF *[50], *HARS *[51], *NCF1 *[52], *UQCRC2 *[53], *ACTG2 *[54], *KCNS3 *[42], *ZHX1 *[55], *EXT1 *[56], *OXR1 *[55], *NDUFS2 *[55], *LMNA *[57], *CCT3 *[58], *PMF1 *[59], *GBA *[60], *PRKAB2 *[61], *S100A6 *[40], *ATP5F1 *[53], *EDG1 *[62], *SLC35A3 *[63], *ATP5B *[44], *EMP1 *[64], *NELL2 *[64], *NFYB *[65], *NFAT5 *[66], *TERF2 *[53], *SIRT2 *[67], *LGALS4 *[67], *GMFG *[67], *FCGRT *[68], *XRCC1 *[27], *FUT1 *[26], *PARK7 *[69], *MAD2L2 *[69], *EIF4G3 *[69], *RPA2 *[69], *TTR *[70], *FUBP1 *[71], *FLOT1 *[72], *BAT1 *[19], *HSPA1L *[19], *ATP6V1G2 *[73], *COL21A1 *[74], *RPS18 *[75], *STK38 *[74], *MTCH1 *[74], *GLO1 *[74], *MUT *[76], *GSTA3 *[74], *GZMH *[19], *CPE *[77], *FGA *[1], *UCP3 *[44], *CRYAB *[44], *SERPINC1 *[78], *F13B *[24], *CTSL2 *[79], *DCTN3 *[80], *PTCHD3 *[81], *GAD2 *[82], *ITIH2 *[83], *AKR1C3 *[84], *HMGB1 *[19], *MRPS31 *[17], *INTS6 *[85], *TOP2B *[86], *MITF *[87], *ARL8B *[19], *CAV3 *[88], *PPARG *[89], *RYK *[90], *PCCB *[86], *RBP2 *[19], *AGTR1 *[40], *SI *[86], *MUC13 *[91], *HCLS1 *[92], *BTG3 *[93], *LPL *[94], *HNRPF *[95], *MYPN *[96], *MBL2 *[97], *MYOM2 *[47], *SLC25A4 *[19], *SNX25 *[10], *STAR *[98], *TTN *[47], *FN1 *[99], *DES *[99], *NNT *[100], *RALY *[101] and *CHRM2 *[102]. The actual positions are given in Additional file 1.

### Validation of the linkage map

After ordering the annotated SNP-containing genes, the homologous genes were used to analyse the known conserved synteny between human and pig of each porcine chromosome [19]. Rearrangements in segments within and between the chromosomes were registered. Single SNP rearrangements were verified on the IMpRH_7000 _panel [12,103] Localisation of the genes was considered according to genes mapped previously. Sequences were analysed by blast [104] against the porcine BAC assembly sequence (pig PreEnsembl, version 20071221142932) [13] to identify potential discrepancies and finally validate marker order.

### Linkage to microsatellite markers

Since this is the first porcine SNP-map, a total of 104 evenly distributed markers from the 18 porcine linkage groups were linked to microsatellites or genes using the whole-genome radiation hybrid IMpRH_7000 _panel. Markers were mapped to the panel comprising 118 hybrid clones (90 clones plus 28 complementary clones) using the IMpRH database [103]. In the two-point analysis the markers were linked to a chromosome by use of the LOD option. The position on the RH map was determined by use of the "linkage of markers to chromosome" option [20]. This mapping facilitates the comparisons of the positions of future QTLs identified by the use of our gene-associated SNP map with results from the whole-genome porcine radiation hybrid map [20].

## Authors' contributions

CB conceived the research and coordinated the project. FP, HH and BZ contributed to annotation of exons, KL to primer design and XW, RKKV, AH and LBM to SNP selection. FP, HH and VRG developed the data pipeline. AH, RKKV and VRG performed the genotyping and VRG validated the data. RKKV and VRG made the linkage groups and RKKV constructed and outlined the linkage maps. BZ, VRG and RKKV annotated the sequences. VRG retrieved reference information for the gene position study, and VRG, RKKV and KKS drafted the manuscript. All authors have read and approved the final manuscript.

## Supplementary Material

Additional file 1 **Gene location.** Genes linked to the microsatellites on the IMpRH_7000_, mapped previously by linkage and physically either by RH panels, somatic-cell hybrids or by Blast analysis of the reference sequence against the pig BAC assembly. The genes are listed in chromosomal (SSC) order. Rearrangements according to the microsatellite map or BAC assembly positions are indicated in bold. Click here for file

Additional file 2 **Data about the SNPs.** The data file contains the following information on each SNP used in the study: SSC number, SNP ID, SNP assays, gene name, sex-averaged distance, female distance, male distance, number of meioses, sow minor allele frequency, number of heterozygous sires, forward and reverse primer sequences, human cytogenetic position, human accession number, physical human location, SNP-containing sequences, SNP status, accession number and name on the PorcineSNP60. Click here for file
